# Ensiling Sorghum with Urea, Aerobic Exposure and Effects on Intake, Digestibility, Ingestive Behaviour and Blood Parameters of Feedlot Lambs

**DOI:** 10.3390/ani13122005

**Published:** 2023-06-16

**Authors:** Maria L. G. M. L. Araújo, Edson Mauro Santos, Gleidson Giordano P. De Carvalho, Douglas dos S. Pina, Juliana S. De Oliveira, Manuela S. L. Tosto, Dayane S. Silva, Alexandre F. Perazzo, Danillo M. Pereira, Thiago Vinicius C. Nascimento, Daniele de J. Ferreira, Hactus S. Cavalcanti, Francisco Naysson de S. Santos, Marinaldo D. Ribeiro, Anderson de M. Zanine

**Affiliations:** 1 Department of Animal Science, Federal University of Bahia, Salvador 40170-110, Brazil; mariaaleonor@hotmail.com (M.L.G.M.L.A.); gleidsongiordano@yahoo.com.br (G.G.P.D.C.); douglaspinaufba@gmail.com (D.d.S.P.); mtosto@ufba.br (M.S.L.T.); dayane.silva40@gmail.com (D.S.S.); 2 Department of Animal Science, Federal University of Paraíba, Areia 58397-000, Brazil; edsonzootecnista@yahoo.com.br (E.M.S.); oliveirajs@yahoo.com.br (J.S.D.O.); 3 Department of Agricultural Planning and Policy, Federal University of Piaui, Teresina 64049-550, Brazil; alexandreperazzo@hotmail.com; 4 Department of Animal Science, Federal University of Maranhão, Chapadinha 65500-000, Brazil; danillomarte.zootec@gmail.com (D.M.P.); hactus_souto@hotmail.com (H.S.C.);; 5 Department of Veterinary Medicine at Sertão, Federal University of Sergipe, Nossa Senhora da Glória 49680-000, Brazil; 6 Department of Animal Science, Federal University of Goiás, Goiânia 74690-900, Brazil; malldorr@gmail.com

**Keywords:** alkaline additive, deterioration, forage conservation, serum metabolites, performance

## Abstract

**Simple Summary:**

Urea, due to its fungicidal effect, when used as a chemical additive in sorghum ensilage improves the aerobic stability of the silage, minimising material losses after opening and exposure to oxygen. Thus, sorghum silage, as a source of roughage, when treated with up to 2% urea based on natural matter, can be used in the feeding of beef sheep in the growth phase, without harming the productive performance of the animals. We also hypothesised that sorghum silages treated with urea, given the potential of this additive, present greater aerobic stability compared to sorghum silages that were not subjected to chemical treatment during ensiling. In this way, it is possible to maintain them for a longer period exposed to oxygen, so that there is little impairment of ingestion, performance and nutritional value of this silage until consumption.

**Abstract:**

This study was carried out to evaluate the effect of ensiling sorghum silage with urea and amending the aerobic exposure nutrients intake and apparent digestibility, ingestive behaviour and blood serum metabolites of feedlot lambs. Forty uncastrated crossbred Dorper × Santa Inês lambs, aged 150 ± 15 days and with an initial body weight of 21.73 ± 2.40 kg, were used. Animals were assigned in a 2 × 3 factorial arrangement. Thus, six silage diets were produced with various urea addition levels (UA: 0 and 5 g/kg on a natural matter basis) and periods of aerobic exposure of silages (PAE: 0, 24 and 48 h). An effect was observed for nutrient intakes of dry matter (DM), organic matter (OM), crude protein (CP), neutral detergent fibre corrected for ash and protein (NDFap) and total digestive nutrients TDN (g/day) and for the total apparent digestibility of DM, OM and CP. There was an interaction effect between urea levels and aerobic exposure for ether extract (EE) and NDFap intakes (g/kg) and nonfibrous carbohydrate (NFC) digestibility (g/kg) (*p* = 0.012). The addition of 5 g/kg of urea to sorghum ensilage improved the digestibility parameters without changing dry matter intake and ingestive behaviour. The addition or not of urea does not change the blood parameters of the animals.

## 1. Introduction

Sorghum (*Sorghum bicolor*, L. Moench), in arid and semi-arid conditions and high altitudes, presents higher growth when compared with corn that promotes greater silage production. The use of this silage in the feed of ruminants is attributed to factors such as lower soil fertility requirements, lower production costs and the possibility of a second cut, as well as greater resistance to drought and high temperatures [1]. However, despite its nutritional value, in some situations when sorghum is ensiled with undesirable characteristics such as a higher content of water-soluble carbohydrates, it is associated with increased acetic acid, or alcoholic silage because of ethanol production, and prone to aerobic deterioration.

The susceptibility to deterioration is a very important factor that determines the quality of silages [2] and, consequently, the dry matter (DM) intake, digestibility and productive performance and health of the animals. A decrease in the nutritive value of silages because of the leaching of some nutrients has been reported to cause an increase in DM, NDF, ADF and ash [2]. Growth of spoilage microorganisms leads to decreases in farm profitability, as the DM losses can reach 20% in corn and sorghum silages. As a result, numerous biochemical, microbiological, physical and management practices have been studied aiming to guarantee the aerobic stability of silages [3].

Silages rich in carbohydrates and well preserved with high concentrations of lactic acid and low levels of volatile fatty acids have been reported to be more prone to aerobic deterioration [4]. Thus, the advantages of ensiling sorghum may be limited by these factors. Therefore, it is necessary to identify suitable additives that inhibit fungi to protect silage after aerobic exposure and promote greater stability of sorghum silage.

Kung Jr. [5] noted that various chemical additives with antifungal properties have been used for aerobic stability of silages, aiming to decrease undesirable fermentation losses. Urea, as a nutrient and aerobic deterioration inhibitor additive [4], should be considered in the ensiling process of sorghum in order to increase the stability of the forage during the feed-out period. Although its antimicrobial mechanisms are not fully elucidated, urea addition during the ensiling process of forages with high contents of water-soluble carbohydrates is an alternative that aims at better control of pH in the silages, as this additive acts to prevent the rapid decrease in pH and development of undesirable microorganisms because of its antimicrobial activity [6]. Additionally, it decreases ethanol production and dry matter and carbohydrate losses through the urease enzyme, as urea is partially converted to ammonia during silage fermentation, and this product has antimicrobial power that inhibits moulds and yeasts, which are responsible for ethanol production. 

Analysing the impact of aerobic exposure of grass silages on dry matter intake and preference in goats, Gerlach et al. [7] concluded that animals are able to detect subtle changes caused by oxygen entry, sometimes even before an increase in temperature or changes in chemical composition occur. The assumption of different authors suggests that unidentified nonvolatile compounds can affect feed preference and intake. However, studies related to urea addition during the ensiling process of sorghum silage and the impact of aerobic exposure of this silage on preference, digestibility of nutrients, ingestive behaviour and blood parameters of lambs are scarce in the literature. 

Although the benefits of urea addition on aerobic stability and chemical composition of silages and hays caused by the improvement of nutritive value of the final product are described, the negative impacts of inclusion on the nutrient intake of animals are also noted. According to Melo et al. [8], the decrease in feed intake noticed after the supply of ammoniated silages is probably due to metabolic factors and/or the palatability of urea. Thus, our hypothesis was that urea addition would increase the aerobic stability of sorghum silages, making them less prone to deterioration, and would result in a better chemical composition in comparison to untreated silages. In addition, it was expected that ensiling sorghum in diets for lambs, with urea (up to 5 g/kg of natural matter) and submitted to up to 48 h of aerobic exposure would not compromise the variables related to ingestive behaviour or blood serum metabolites, resulting in better performance. Thus, this study was carried out to evaluate the influence of increasing periods of aerobic exposure of sorghum silages, treated or not with urea, on nutrient intake, total apparent digestibility of nutrients, ingestive behaviour and blood serum metabolites of feedlot lambs.

## 2. Materials and Methods

This study was conducted in strict accordance with the recommendations in the Guide for the National Council for the Control of Animal Experiments (CONCEA). The protocol was approved by the Committee on the Ethics of Animal Experiments of the Federal University of Bahia (UFBA), Salvador, Brazil (protocol number 86/2017).

### 2.1. Silage Production and Diets 

Sorghum (*Sorghum bicolor* (L). Moench, Volumax hybrid) was planted using a direct planting system, with 70 cm between rows conserving 12 plants per linear metre, at the Experimental Farm of São Gonçalo dos Campos belonging to the Federal University of Bahia (12°23′49.5″ S and 38°52′43.5″ W, altitude: 234 m). The climate is classified according to the Köppen–Geiger classification as ‘As’ (‘A’ tropical climate, with average temperatures above 18° and ‘s’, dry season occurs when the days are longer), with average annual precipitation of 900 to 1200 mm per year.

Fertilisation was performed according to the soil analysis and crop demands, with 300 kg of phosphate super simple, 100 kg of potassium chloride and 200 kg ha^−1^ of urea. Plants were harvested mechanically after 110 days, when the grains had a dough-to-dent consistency, with approximately 30–35% of DM and chopped in a forage chopper coupled to a tractor with the particle size adjusted for a theoretical cut length of approximately 5 mm. 

After obtaining the above chopped material and before the silage-making process, the sorghum was transported and mixed with urea at proportions of 0 and 5 g/kg (on a natural matter basis) in the mixture. Then, the material was compacted using a tractor, placed in ‘surface silos’, sealed with black-on-white polyethylene film (thickness = 200 µm), which was also used to avoid the contact of forage with soil, and then stored in the field for 150 days. 

### 2.2. Animal Study, Location of the Experiment and General Procedures

The animal study experiment, crop harvesting and ensiling were conducted at the Experimental Farm of the Federal University of Bahia located at São Gonçalo dos Campos, School of Veterinary Medicine, State of Bahia, Brazil: 12°23′49.5″ S latitude and 38°52′43.5″ W longitude.

Forty uncastrated crossbred five-month-old Dorper × Santa Inês lambs weighing 21.73 ± 2.40 kg (mean ± standard deviation) were used. Animals were kept individually in covered stalls (1.0 × 1.0 m^2^) with a slatted and suspended floor, equipped with drinking fountains and feeders, with ad libitum access to water and feed during the experimental period.

During the adaptation period, animals were identified, vaccinated against clostridia and treated to control internal and external parasites. The experiment was conducted over a 25-day period, which was preceded by 10 days of the animals’ adaptation to management and diets and 15 days for data collection. 

The experimental design adopted to evaluate the urea addition and aerobic exposure of silages was completely randomised in a 2 × 3 factorial arrangement consisting of a treatment without addition of urea (control) and 5 g/kg of urea in sorghum silage (on a natural matter basis) and three periods of aerobic exposure of silages (0, 24 and 48 h).

Lambs were fed a total mixed ratio, twice a day (09:00 and 16:00 h), composed of 500 g/kg of sorghum silage (untreated and treated with 5 g/kg of urea submitted to 0, 24 and 48 h of aerobic exposure) and 500 g/kg of a concentrate that was constituted of ground corn, soybean meal, mineral premix and urea (Table 1 and Table 2).

Diets were formulated based on to the recommendations of the National Research Council (2007) [12] and contained 14% of CP per kg of DM to meet the nutritional requirements of lambs with an estimated average daily gain (ADG) of 200 g/day.

### 2.3. Sample Collection and Laboratory Analysis

During the experimental period, samples of silages, feeds (ingredients and diets), leftovers and faeces were collected, pre-dried in a forced-ventilation oven at 55 °C for 72 h and ground in a Willey knife mill (Tecnal^®^, Piracicaba City, São Paulo State, Brazil) using a 1 mm sieve. Then, they were sealed in airtight plastic containers until later laboratory analysis to determine dry matter (DM—method 967.03), mineral matter (MM—method 942.05), crude protein (CP—method 981.10) and ethereal extract (EE—method 920.39) according to the Association of Official Analytical Chemists [13].

Neutral detergent fibre (NDF) was determined with heat stable amylase without sodium sulphite, according to Detmann et al. [14]. The acid detergent fibre (ADF) content was determined as described by Van Soest et al. [15]. The acid detergent lignin (ADL) content was determined using method 973.18 of the AOAC [13], by which fibre residue is treated with 72% sulphuric acid. The neutral and acid detergent digestion residues were corrected for ash and protein, being incinerated in a muffle oven at 600 °C for four hours, and the protein correction was based on neutral detergent insoluble protein (NDIP) and acid detergent insoluble protein (ADIP), according to Licitra et al. [16]. 

Nonfibrous carbohydrates (NFC) were estimated according to the methods described by Mertens [17], considering the value of NDF corrected for ash and protein = 100 − %CP + %NDFcp + %EE + %ash. 

The other cell wall fractions were determined using the equations described by Van Soest et al. [15]: hemicellulose = NDF − ADF, and cellulose = ADF − ADL. Total digestible nutrient (TDN) contents of the ingredients and silages (Table 1) were calculated using the equations of Detmann et al. [9,10,11] regarding the contents of apparently digestible fractions of NFC, ether extract, crude protein and neutral detergent fibre corrected for ash and protein for finishing cattle.

### 2.4. Nutrient Intake and Total Apparent Digestibility

After the fermentation period (150 days), prior to being supplied to the animals, silos were opened and the amount of daily silage from each treatment (silages treated with 0 and 5 g/kg of urea) was exposed to oxygen in order to be submitted to different periods of aerobic exposure (from 0 to 48 h). Silages were maintained in contact with the concrete floor of the covered shed, in the same place that animals were allotted. Thus, both untreated and treated silages (0 and 5 g/kg of urea, on a NM basis) with zero hours of exposure to air were taken in higher amounts in order to ensure that it would be available to be offered to lambs with periods of 24 and 48 h of aerobic exposure.

Nutrient intake was estimated by subtracting the amount of each nutrient contained in the refused feed from the total of each nutrient in the offered feed, being expressed in grams per day (g/day) and grams per kilogram of body weight (g/kg BW). Adjustments were made to ensure that the leftovers were between 10–20% so that there was no dietary restriction on the animals.

The digestibility assay was performed over five consecutive days of the experimental period. For the collection of faeces, appropriate bags were attached to the animals for total faecal collection. After a period of three days of adaptation to the collection bags, two subsequent days were dedicated to faecal collection twice a day (at 08:00 and 15:00 h). A 10% aliquot of the total amount of faeces collected was stored in individual plastic bags, identified and stored in a freezer at −20 °C. Additionally, during the trial, samples of ingredients and leftovers of each animal were collected and stored in plastic bags in the same freezer until the analyses.

The samples were dried in a forced-ventilation oven at 55 °C for 72 h and ground in a Willey knife mill (Tecnal^®^, Piracicaba City, São Paulo State, Brazil) using a 1 mm sieve, then used for chemical analyses as previously mentioned. 

The apparent digestibility coefficients (DCs) of dry matter, crude protein, neutral detergent fibre and ether extract were calculated as follows:

$$DC=\frac{\left(kg\;of\;nutrient\;portion\;ingested-kg\;of\;nutrient\;portion\;excreted\right)}{\left(kg\;of\;the\;nutrient\;portion\;ingested\right)}\times 100$$

The intake of total digestible nutrient (TDN) was calculated according to Weiss et al. [18]. 

### 2.5. Ingestive Behaviour Evaluation

Ingestive behaviour was observed over 24 h (beginning at 09:00 h, after the diet was offered to the animals) at intervals of 15 min to evaluate time spent on feeding, rumination and idling, expressed in minutes/day [19]. Data on each animal’s behavioural activities were recorded by two trained observers, who were arranged so as to minimise interference in the animals´ behaviours. Observers took turns every three hours, and during the night-time, the evaluations were conducted with the environment maintained with artificial lighting. 

The animals’ daily behavioural activities were divided into three periods: morning, afternoon and night. Each animal was observed in three moments in each period. In these moments, the number of chews from each ruminal bolus per animal and the time spent for rumination of each bolus were recorded, with the aid of digital chronometers. Based on these three measurements, the mean parameters were calculated for each animal for statistical analysis. The data for behavioural variables were obtained according to the methodology described by Burger et al. [20]. 

Samples of silage, concentrate and leftovers of each animal after the ingestive behaviour evaluations were collected, conditioned in plastic bags, properly identified and stored in a freezer at −20 °C and later submitted to laboratory analyses for the estimation of the intake and efficiencies of feeding and rumination.

### 2.6. Blood Sample Collection and Metabolites Analysed

Before feeding, blood samples (10 mL) from each animal were collected by puncturing the jugular vein using vacutainer tubes containing anticoagulant (EDTA—ethylenediamine tetra acetic acid). Samples were kept at room temperature until clot retraction. Then, they were centrifuged at 3500 rpm for 15 min and the serum was stored in 2 mL Eppendorf tubes and frozen at −20 °C for further analysis of blood metabolites.

Serum total protein and albumin serum concentrations were determined by the biuret method and bromocresol green solution using commercial kits (Doles, Goiânia, Brazil—total proteins, albumin). The globulin content was calculated by the mathematical difference between total protein content and serum albumin, with values expressed in g/dL. The albumin:globulin ratio was obtained by dividing the value of the albumin fraction by the total value of the globulin fraction. 

Serum urea levels were determined by the kinetic system using a commercial kit (Doles, Goiânia, Brazil—Ureia UV) and creatinine by a colorimetric system using a commercial kit (Doles, Goiânia, Brazil—Creatinine). 

Serum cholesterol and triglyceride concentrations were determined by the enzymatic technique using commercial kits (Doles, Goiânia, Brazil—liquid enzyme cholesterol and liquid enzyme triglycerides). Enzyme activities for liver metabolism, alanine aminotransferase (ALT), aspartate aminotransferase (AST) and gamma-glutamyl transferase (GGT) were measured by the kinetic system using commercial kits (Doles, Goiânia, Brazil—ALT/TGP; AST/TGO and γ-glutamyl transferase). Concentrations of blood metabolites were read using a semi-automatic spectrophotometer (SBA 200^®^, CELM, São Caetano do Sul, Brazil) according to their respective wavelengths.

### 2.7. Statistical Analyses

The animals were distributed in a completely randomised experimental design with six treatments, arranged in a 2 × 3 factorial scheme: two sorghum silage additives (sorghum without urea addition and sorghum ensiled with 5 g/kg urea, on a natural matter basis) and three periods of aerobic exposure of the silage (0, 24 and 48 h).

Nutrient intake, digestibility coefficient, ingestive behaviour and concentrations of blood metabolites were submitted to analysis of variance (ANOVA) according to the statistical model described below:

Y*ijk* = µ + S*i* + T*j* + S*i* × T*ij* + e*ijk*

where μ = overall mean; S*i* = fixed effect of urea addition levels during sorghum ensiling (1–2); T*j* = fixed effect of periods of aerobic exposure (1–3); *Si* × T*ij* = effect of the interaction between silage (S) and periods of aerobic exposure (PAE); e*ijk* = random error, normal and independently distributed with a mean of zero and variance of δ^2^.

The initial body weight was considered as a covariate for the performance variables. Least square means were compared by the Tukey test, considering a 5% probability level of a type-I error. Statistical analyses were performed using PROC MIXED in SAS software 9.0 [21].

## 3. Results

### 3.1. Nutrient Intake and Digestibility Coefficients 

The intakes of DM, OM, CP, NFC and TDN, expressed in kg/day, were affected only by the aerobic exposure (*p* < 0.05). Thus, the intake values of these nutrients at 48 h were lower than at 0 and 24 h of aerobic exposure (*p* < 0.05), but the values of intake of these nutrients at 0 and 24 h of aerobic exposure did not differ (*p* > 0.05) (Table 3). The intake of DM (g/kg BW) and apNDF (kg/day) were affected by the urea addition level in sorghum silage (*p* = 0.039 and 0.004, respectively) and the period of aerobic exposure (*p* = 0.006 and 0.022, respectively). The highest values for these intakes were observed for animals fed with sorghum silage without urea addition in comparison to sorghum silage treated with 5 g/kg of urea (Table 3).

The same effects were observed in the intake of these nutritional fractions relative to the effect of the period of aerobic exposure, in which animals fed with silage submitted to zero hours of aerobic exposure presented a higher intake (*p* < 0.05), differing from those fed the silage with 48 h of aerobic exposure. However, the intake of the animals fed the silage with 24 h of exposure did not differ (*p* > 0.05) from the animals fed with silage in both treatments.

The intake of ether extract (kg/day) (*p* = 0.006) and apNDF (g/kg BW) (*p* < 0.0001) showed an interaction effect between the urea addition level in sorghum silage and periods of aerobic exposure (Table 3).

The digestibility of DM, OM, CP and NFC was affected by both urea addition level (*p* = 0.0214; *p* = 0.0132; *p* = 0.0002; *p* = 0.0001, respectively) and the periods of aerobic exposure (*p* = 0.048; *p* = 0.049; *p* = 0.0001; *p* = 0.028, respectively) (Table 3). Additionally, the digestibility of apNDF was not influenced by the urea treatment of sorghum (*p* = 0.844), nor by the time of aerobic exposure (*p* = 0.205) (Table 3). 

Regarding the effect of the aerobic exposure times, it was observed that only the digestibility of EE and apNDF were not affected with the time of aerobic exposure (*p* > 0.05). These variables did not differ (*p* > 0.05) when compared with the urea addition level and the interaction between urea addition level and aerobic exposure times (*p* > 0.05) (Table 3).

In relation to the digestibility of the total digestible nutrients, only effects (*p* = 0.001) of the urea addition level in the silage offered to the animals were verified, with the highest values observed for the sorghum silage treated with 5 g/kg urea (Table 3).

Animals fed with silage without urea addition (0 g/kg) and submitted to aerobic exposure for 48 h showed the lowest ether extract intake compared to the time of 48 h (*p* = 0.006) (Table 4). Regarding silage with a level of 5 g/kg urea, that submitted to aerobic exposure for 0 h showed the highest ether extract intake (Table 4). 

Animals fed with the control silage (without the addition of urea) and submitted to aerobic exposure for 24 h showed the highest intake of apNDF (g/kg BW) (*p* = 0.006). Thus, the lowest intake of apNDF was observed in the aerobic exposure time of 48 h combined with 5 g/kg urea (*p* < 0.001) (Table 4).

However, with zero hours of aerobic exposure, no effect (*p* > 0.05) of urea addition in sorghum silage on nonfibrous carbohydrate digestibility was observed; the opposite was observed at 24 and 48 h of exposure. Therefore, silages treated with 5 g/kg urea had higher digestibility values of nonfibrous carbohydrates compared to silages without the use of the additive (Table 4).

### 3.2. Ingestive Behaviour 

In general, intake of DM and apNDF for 24 h (g/day), as well as feeding, rumination, idling and total chewing times expressed in minutes per day, were not influenced (*p* > 0.05) by urea addition, aerobic exposure times and the interaction between the analysed factors (Table 5).

Ingestive behaviour variables related to feeding and rumination efficiencies, expressed in grams of DM and NDFD per hour and grams of DM and NDFD per bolus, were not affected (*p* < 0.05) by urea addition in sorghum silage, nor by the periods of aerobic exposure (Table 5). 

### 3.3. Blood Metabolites 

In general, the blood metabolites of lambs fed with the treated silage did not show a significant effect (*p* > 0.05) of urea addition, aerobic exposure or interaction times on the variables of serum creatinine, total protein, albumin, globulin and albumin: globulin (A:G). However, there was an effect of serum urea (*p* = 0.043) on plasma urea levels, with higher mean values in animals fed with sorghum silage with the addition of 5 g/kg urea (Table 6).

The serum concentrations of cholesterol, triglycerides and aspartate aminotransferase (AST) did not differ (*p* > 0.05) because of the types of silage or the aerobic exposure periods evaluated. However, the ALT and GGT serum activities were influenced by the exposure period and the types of silage, respectively (Table 6).

## 4. Discussion

The lambs fed with silage exposed for 48 h had a lower intake of dry matter regardless of the addition of urea. Possibly, due to the reduced digestibility of the silage following aerobic exposure for 48 h, the animals had a higher emptying of the gastrointestinal tract, limiting the feed intake. 

Silage quality can be influenced by factors such as the stage of development of the crop at the time of cutting, compaction and sealing and by the fermentative processes, as well as by the exposure to the oxygen to which it is submitted. According to Reis et al. [22], during silage fermentation, changes in nutritive value occur, resulting in a decrease in soluble carbohydrate and protein contents associated with an increase in the concentration of organic acids and nonprotein nitrogen. Weiss et al. [18] stated that the ingestion of silage is not similar compared to the intake of the original forage. Based on the findings of these authors, it is inferred that aerobic exposure reduces dry matter digestibility because of decreases in the digestion of dry matter, causing a decrease in the rate of passage. Another possible explanation may be the oxidation of organic compounds causing an unpleasant odour, flavour and taste that inhibits the ingestion of silage.

Aerobic stability is an important tool used for the analysis of silage quality. Therefore, when the silage is opened, the deterioration process starts, promoting a decrease in the nutritional value of the forage because of fermentation product losses, which arise from the potentially digestible substrates [23]. Several factors in association will determine the aerobic stability of the silage, which may deteriorate more quickly or slowly depending on the fermentation products [3,5].

The lower intake might therefore be a result of both sensory characteristics, principally smell and a negative post-ingestive feedback derived from higher amine concentrations from the urea hydrolyzation as these higher levels of volatile nonprotein nitrogen have a bitter taste or other unidentified silage characteristics [24].

Pahlow et al. [25] noted that the aerobic deterioration of silage is mainly related to the development of yeasts, fungi and aerobic bacteria (bacilli). Thus, in general, this process has its beginning because of the yeasts, which act through the oxidation of sugars and lactic acid to form carbon dioxide and water. According to Reis et al. [22], in addition to sugars, other substrates are used by deteriorating microorganisms, such as acids and proteins, resulting in an increase in pH and an associated decrease in digestibility and energy content.

Different from the observed nutrient intake behaviour, digestibility was generally influenced by the addition of urea to the sorghum silage. Therefore, the nutrient fraction digestibility, except for EED and apNDFD, did not affect the addition of urea and the time at which silages were submitted to aerobic exposure (Table 3). 

On the other hand, when the silage was exposed to oxygen for up to 48 h, a reduction in the nutrient digestibility was observed, which may have occurred because, when the silage is oxygen exposed, this causes the proliferation of fungi that consume lactic acid, protein and nonfibrous carbohydrates, reducing the nutritional value of the silage and transforming the nutritional fractions into carbon dioxide, water and volatile compounds with a strong odour. 

The literature reports that the use of hydrolytic agents in the chemical treatment of forages, such as anhydrous ammonia, ammonium hydroxide and urea as a source of ammonia, is capable of causing changes in the fibrous fraction of the cell wall, increasing the digestibility of apNDF caused by the solubilisation of part of the hemicellulose and by the expansion of cellulose [24]

Thus, there is a reduction in the NDF content, as the hydrogen bonds are broken, and there is an increase in the hydration of the fibre, thus allowing the access of the microorganisms. However, the effect of urea on forage, the efficiency of using urea in silage, depends on factors such as the applied dose and the forage storage time, so the beneficial effects of ammonia use on forage may not be observed [2].

Although there was an interaction in the intake of ether extract of the animals, the digestibility of this fraction did not follow the same path. As all the animals were fed a roughage:concentrate ratio of 50:50, and no ingredients containing different energy densities were included in the diets, it would not be expected that there would be changes in the intake of this fraction and, in turn, in the digestibility.

Regarding the digestible nutrients, only the NFC showed a combined effect of additives in silage and duration of aerobic exposure. A positive effect of the addition of urea on NFC digestibility was observed. Probably, the presence of urea limited yeast proliferation after exposure to oxygen, which increased the stability of the silage after aerobic exposure.

As mentioned by Woolford [26], during the silage period, due to the cleavage of the forage, the release of intracellular content such as protein, NFC and enzymes occurs because of the rupture of the cells. Thus, the microbial population has access to intracellular content, and simultaneously, the population and enzymes present in the plant perform the hydrolysis of proteins, resulting in the production of peptides and free amino acids, as well as the conversion of complex carbohydrates into simple sugars. Possibly, the increase in NPN contents caused by the use of urea and the changes in the NDF contents of the diets allowed the additions in the digestibilities of the fractions in the treated silages. Thus, solubilisation of part of the hemicellulose possibly increased the availability of fermentable substrates, providing adequate conditions for greater microbial development, which resulted, in general, in higher digestibility of the treated fodder.

The results obtained in this study corroborate those of Schlatter and Smith [27], which stated that changes in silage can decrease the nutrient concentration fraction, negatively affecting feed digestibility. In a similar way, Reis et al. [22] also mentioned that the nutritional value of silage is first defined by the digestibility of the nutrients, which is a direct effect of the fermentation pattern and deterioration processes observed during the aerobic phase.

Kung Jr. [5] stated that urea, as an additive hydrolysed by the action of urease, promotes the release of ammonia (NH_3_). Thus, the decrease in the pH value is milder and extends the fermentation time, which is associated with inhibition of the growth of secondary fermentation bacteria, causing greater use of the fermentable substrates of the ensiled material, affecting the final quality of the silage.

Despite the small variation in the chemical composition of the silages and experimental diets, these differences were not enough to promote a significant effect on the intake of DM and apNDF (grams/day) by the animals.

The efficiencies of feeding and rumination of DM and apNDF were also not influenced by the levels of urea inclusion or the periods of aerobic exposure of the sorghum silages, probably because of the similarities of DM intake. Thus, it can be concluded that the inclusion of up to 5 g/kg of urea associated with periods of up to 48 h of aerobic exposure of sorghum silages were not sufficient to promote changes in the ingestive behaviour of animals. This result corroborates with Carvalho et al. [19], who mentioned that efficiencies of feeding and rumination are mainly affected by the intake of the animals. As a result, it affects the time spent in feeding, rumination and idling, and it is also important to evaluate the quality of feed with low digestibility. 

Among the blood metabolites related to the protein profile, only the serum urea concentrations were affected by the urea addition. Thus, animals fed silages treated with 5 g/kg of urea had higher values of urea compared to those fed control silages (without the additive). Although there was no effect of the aerobic times on this variable, all the animals showed mean values higher than the levels of normality described by Kaneko et al. [22] for ovine species. Thus, this change in serum urea values probably occurred because of the inclusion of the additive as a source of nonprotein nitrogen.

The true protein fraction of the silage, according to Van Soest [28], can represent 60–80% of the nitrogen in the forage, and the remaining total corresponds to the nonprotein and unavailable nitrogen. The serum urea concentrations have a direct association with protein intake; diets may have caused a higher CP supply or an inadequate energy/protein ratio, resulting in increases in ammonia in the rumen, as evidenced by the increase in serum urea levels.

The different periods of aerobic exposure of sorghum silages associated with or without 5 g/kg of urea had no influence on this variable. Thus, lambs may still be at a stage of development that would allow mobilisation of muscle tissue, justifying this result.

Considering that the albumin concentration was not altered for the animals fed silages with urea added, it is inferred that the lambs were not in a nutritional deficiency state, since albumin is a serum component indicative of compliance with the protein nutritional requirements for animal maintenance. Serum protein is composed of albumin and globulin, the latter reported as having less dietary influence on these compounds, which in turn is commonly associated with the animal’s immune status (physiological stress marker or chronic infections) and the occurrence of liver disorders, such as acute or chronic hepatitis. In this study, values of albumin and globulin were found to be within normal levels for the ovine species.

The urea addition or the duration of aerobic exposure did not modify the serum concentrations of albumin and globulin, nor the albumin:globulin ratio. 

In the present study, cholesterol and triglyceride values were not affected by the addition of urea to the sorghum silage or by the periods of aerobic exposure. These serum variables are altered by the ingestion of lipids; thus, as the variation in the lipid content of the diets was low, it did not cause any alteration in these variables. Therefore, although diets and silages have similar ether extract composition, there was a change in their consumption with the addition of urea and with aerobic exposure, but this difference maintained a similarity in dietary lipid intake resulting in a similar availability of absorbed fatty acids, which caused similar serum concentrations of cholesterol and triglycerides.

Serum liver enzyme activities were within proposed normal levels for sheep. In general, changes in liver enzymes are due to intense asynchrony in protein and energy intake, causing accumulation of liver fat. Other probable causes for changes in these parameters are liver diseases or tissue damage in the liver. However, according to the authors, ALT activities that should have been between 26–34 IU/L were higher in all animals, which may be related to sudden changes in weight, and it is possible to suggest that the diets caused some hepatic tissue damage, as this enzyme commonly exhibits increased values when hepatocyte membrane integrity is impaired.

The authors should discuss the results and how they can be interpreted from the perspective of previous studies and of the working hypotheses. The findings and their implications should be discussed in the broadest context possible. Future research directions may also be highlighted.

## 5. Conclusions

The addition of 5 g/kg of urea to sorghum ensilage improved digestibility parameters without changing dry matter intake and ingestive behaviour. However, the longer period of aerobic exposure caused lower consumption and digestibility, and the addition or not of urea does not change the blood parameters of the animals.

## Figures and Tables

**Table 1:** Chemical composition of ingredients used in the experimental diets fed to crossbreed Santa Inês lambs.

Item	Ground Corn	Soybean Meal	Urea Level					
			(0 g/kg Urea NM)			(5 g/kg Urea NM)		
			Aerobic Exposure (h)					
			0	24	48	0	24	48
Dry matter (g/kg)	903.0	914.9	258.1	266.9	269.1	213.8	212.7	211.5
Ash (g/kg DM)	16.6	75.4	51	52.5	46.2	65.1	66.3	57.3
Crude protein (g/kg DM)	81.6	468.9	80.7	76.5	71.7	110.7	113.7	121.2
Ether extract (g/kg DM)	41.1	20.8	17.3	19.1	12.5	46.7	30.9	25.7
Neutral detergent insoluble protein (g/kg CP)	101.8	59.7	66.2	62.8	69.5	80.5	54.5	31.7
Acid detergent insoluble protein (g/kg CP)	152.2	143.3	202.8	194.1	226.1	206.3	257.9	183.1
Neutral detergent fibre (g/kg DM)	131.9	113.2	621.1	589.8	583.3	556	540.4	528.2
Acid detergent fibre (g/kg DM)	38.0	78.7	401.3	394.7	382.1	423.6	379.8	387.9
Lignin (g/kg DM)	9.2	5.8	67.7	66.7	65.5	80.7	66.5	70.5
Cellulose (g/kg DM)	28.8	72.9	333.6	328.0	316.6	342.9	313.3	317.4
Hemicellulose (g/kg DM)	93.9	34.5	219.8	195.1	201.2	132.4	160.6	140.3
Nonfibrous carbohydrates (g/kg DM)	728.8	321.7	229.9	262.1	286.3	221.5	248.7	267.6

Natural matter.

**Table 2:** Ingredients and chemical composition of the experimental diets containing sorghum silage submitted to aerobic exposure (0, 24 and 48 h) and fed to crossbreed Santa Inês lambs.

Ingredients Proportions (g/kg DM)	^1^ Urea Addition Level (g/kg NM Basis)					
	0			5		
Ground corn	385			380		
Soybean meal	100			100		
^2^ Mineral premix	15			15		
Urea	0			5		
Sorghum silage	500			500		
Chemical composition of diets						
Analytical fraction	Urea level					
	(0 g/kg urea NM basis)			(5 g/kg urea NM basis)		
	Aerobic exposure (hours)					
	0	24	48	0	24	48
Dry matter (g/kg as fed)	583.7	588.1	589.2	561	560.5	559.9
Ash (g/kg DM)	33.8	60.1	56.9	54.4	55.2	52.0
Crude protein (g/kg DM)	132.3	130.2	137.8	133.7	135.2	138.9
Ether extract (g/kg DM)	26.3	27.2	23.9	26.6	27.5	24.2
Neutral detergent insoluble protein (g/kg CP)	125	123.3	126.6	133.2	120.2	108.8
Acid detergent insoluble protein (g/kg CP)	173.6	169.2	185.2	176.1	201.9	164.5
Neutral detergent fibre (g/kg DM)	372	356.3	353.1	340.1	332.3	324.9
Acid detergent fibre (g/kg DM)	223	219.7	213.4	234.3	212.4	218.6
Lignin (g/kg DM)	37.9	37.4	36.8	44.5	37.4	39.4
Cellulose (g/kg DM)	185.1	182.3	176.6	189.8	175.0	179.2
Hemicellulose (g/kg DM)	149	136.6	139.7	105.8	119.9	106.3
Nonfibrous carbohydrates (g/kg DM)	424.1	440.2	452.3	423.5	437.1	446.6
^3^ Total digestible nutrients (g/kg)	699.3	673.8	648.3	711.8	672.3	653.3

^1^ Natural matter; ^2^ guaranteed levels (per kg, in active elements): 120.00 g calcium, 87.00 g phosphorus, 147.00 g sodium, 18.00 g sulphur, 590.00 mg copper, 40.00 mg cobalt, 20.00 mg chromium, 1800.00 mg iron, 80.00 mg iodine, 1300.00 mg manganese, 15.00 mg selenium, 3800.00 mg zinc, 300.00 mg molybdenum, 870.00 mg maximum fluoride, solubility of phosphorus (P) in citric acid minimum 2–95%; ^3^ calculated from the methodology of Detmann et al. [9,10,11].

**Table 3:** Daily nutrient intake (expressed in kg and g/kg BW) and apparent digestibility coefficient of lambs fed sorghum silage treated with urea submitted to increasing periods of aerobic exposure (0, 24 and 48 h).

Item	^1, 2^ Urea Level (g/kg NM)		^3^ Aerobic Exposure (h)			^4^ SEM	^5^ *p*-Value		
	0	5	0	24	48		UA	PAE	^6^ UA × PAE
Intake									
Dry matter (kg/day)	0.81	0.78	0.87a	0.82a	0.70b	0.039	0.419	0.011	0.402
Dry matter (g/kg BW)	28.39	26.81	29.07a	27.92a	25.90b	0.736	0.039	0.006	0.369
Organic matter (kg/day)	0.76	0.73	0.82a	0.77a	0.65b	0.041	0.448	0.011	0.433
Crude protein (kg/day)	0.11	0.11	0.12a	0.12a	0.10b	0.005	0.422	0.010	0.683
Ether extract (kg/day)	0.02	0.03	0.03a	0.03a	0.02b	0.001	<0.0001	0.0001	0.006
^8^ apNDF (kg/day)	0.27	0.23	0.28a	0.26ab	0.23b	0.012	0.010	0.022	0.818
^7, 8^ apNDF (g/kg BW)	8.77	7.93	8.36a	8.79a	7.92b	0.242	0.004	0.042	<0.001
Nonfibre carbohydrates (kg/day)	0.37	0.38	0.40a	0.39a	0.34b	0.018	0.457	0.041	0.383
Total digestible nutrients (kg/day)	0.56	0.59	0.64a	0.58a	0.51b	0.032	0.390	0.026	0.908
Digestibility coefficient (kg/kg DM)									
Dry matter	0.674	0.713	0.722a	0.688ab	0.672b	0.0148	0.021	0.048	0.294
Organic matter	0.681	0.723	0.730a	0.696ab	0.681b	0.0146	0.013	0.049	0.312
Crude protein	0.834	0.773	0.843a	0.803b	0.764c	0.0142	0.001	<0.001	0.237
Ether extract	0.780	0.819	0.831	0.775	0.791	0.0206	0.083	0.102	0.569
apNDF ^§^	0.442	0.447	0.472	0.452	0.409	0.0265	0.844	0.205	0.883
Nonfibre carbohydrates	0.824	0.872	0.872a	0.829c	0.844b	0.0119	0.001	0.028	0.012
Total digestible nutrients	0.683	0.733	0.732	0.699	0.693	0.0125	0.001	0.058	0.298

^1^ Natural matter; ^2^ urea addition level (on natural matter basis); ^3^ periods of aerobic exposure; ^4^ standard error of the mean; ^5^ means followed by different lowercase letters (a, b, c) differ statistically (*p* < 0.05) according to Tukey’s test; ^6^ interaction effect between urea addition level and periods of aerobic exposure; ^7^ body weight; ^8^ neutral detergent fiber corrected for ash and protein ^§^ corrected ash and protein.

**Table 4:** Decomposition of the interactions for productive variables of lambs fed sorghum silage treated with urea submitted to increasing periods of aerobic exposure (0, 24 and 48 h).

Item	^1^ Urea Level (g/kg NM)	Aerobic Exposure (h)		
		0	24	48
Ether extract intake (kg/day)	0	0.023Ba	0.024Aa	0.019Ab
	5	0.037Aa	0.028Ab	0.022Ac
^2, 3^ Intake of apNDF (g/kg BW)	0	7.77Bc	9.92Aa	8.61Ab
	5	8.92Aa	7.65Bb	7.22Bb
^4^ Digestibility of nonfibre carbohydrates	0	0.877Aa	0.794Bb	0.803Bb
	5	0.867Aa	0.864Aa	0.885Aa

^1^ Natural matter; ^2^ ash and protein corrected neutral detergent fibre; ^3^ body weight; ^4^ nonfibrous carbohydrates; lowercase and uppercase letters correspond to rows and columns, respectively. Means followed by different letters differ statistically (*p* < 0.05) according to Tukey’s test.

**Table 5:** Ingestive behaviour of lambs fed sorghum silage treated with urea submitted to increasing periods of aerobic exposure (0, 24 and 48 h).

Item	^1, 2^ Urea Level (g/kg NM)		Aerobic Exposure (h)			^3^ SEM	^4^ *p*-Value		
	0	5	0	24	48		UA	PAE	^5^ UA × PAE
Intake in 24 h (grams/day)									
^6^ Dry matter	811.29	851.04	816.23	871.74	805.53	46.31	0.476	0.543	0.991
^7^ Neutral detergent fibre_ap_	578.99	590.95	633.51	568.45	552.95	54.86	0.779	0.231	0.940
Time spent (minutes/day)									
Feeding	210.53	195.56	204.65	195.07	209.40	7.48	0.126	0.462	0.211
Rumination	506.50	539.17	533.82	539.06	495.62	22.37	0.256	0.403	0.609
Idling	717.49	713.75	701.53	717.92	727.41	24.31	0.903	0.785	0.276
^8^ Total chewing time (hours/day)	12.04	12.10	12.30	12.03	11.87	0.40	0.904	0.786	0.275
Feeding efficiency (grams/hour)									
Dry matter	236.23	261.50	247.27	272.95	226.38	16.15	0.213	0.172	0.404
Neutral detergent fibre_ap_	165.17	193.65	188.40	176.84	172.98	19.65	0.077	0.686	0.806
Rumination efficiency (grams/hour)									
Dry matter	100.51	95.23	96.73	97.57	99.31	7.130	0.560	0.972	0.697
Neutral detergent fibre_ap_	71.14	62.28	71.60	66.23	62.29	8.306	0.185	0.520	0.994
Rumination efficiency (grams/bolus)									
Dry matter	1.54	1.46	1.52	1.45	1.53	0.139	0.685	0.908	0.131
Neutral detergent fibre_ap_	0.89	0.81	0.87	0.85	0.84	0.102	0.342	0.970	0.924

^1^ Natural matter; ^2^ urea addition level (on natural matter basis); ^3^ standard error of the mean; ^4^ means followed by different lowercase letters differ statistically (*p* < 0.05) according to Tukey’s test; ^5^ interaction effect between urea addition level and periods of aerobic exposure; ^6^ dry matter; ^7^ neutral detergent fibre corrected ash and protein; ^8^ total chewing time = includes eating and rumination activities.

**Table 6:** Blood metabolites of lambs fed sorghum silage treated with urea submitted to increasing periods of aerobic exposure (0, 24 and 48 h).

Item	^1, 2^ Urea Level (g/kg NM)		Aerobic Exposure (h)			^3^ SEM	^4^ *p*-Value		
	0	5	0	24	48		UA	PAE	^5^ UA × PAE
Proteic Profile									
Urea (mg/dL)	45.17	50.83	50.51	48.00	45.50	2.441	0.043	0.315	0.928
Creatinine (g/dL)	0.151	0.146	0.151	0.148	0.146	0.016	0.784	0.928	0.641
Total protein (g/dL)	6.35	6.05	6.20	5.91	6.50	0.229	0.241	0.174	0.313
Albumin (g/dL)	2.49	2.78	2.61	2.54	2.75	0.142	0.071	0.528	0.394
Globulin (g/dL)	3.80	3.46	3.87	3.37	3.66	0.266	0.247	0.374	0.341
^6^ Albumin: globulin ratio	0.67	0.81	0.81	0.76	0.65	0.073	0.089	0.308	0.502
Energetic profile (mg/dL)									
Cholesterol	74.77	71.56	77.97	71.65	69.87	5.286	0.575	0.475	0.465
Triglycerides	41.55	38.80	40.27	41.82	38.43	3.421	0.430	0.697	0.245
Enzymatic profile (IU/L)									
^7^ Aspartate aminotransferase	65.63	65.41	63.31	65.44	67.81	6.561	0.976	0.874	0.412
^8^ Alanine aminotransferase	43.36	42.73	42.56b	42.54b	44.04a	0.497	0.232	0.034	0.223
^9^ Gamma-glutamyl transferase	44.02	52.33	45.25b	47.67b	51.61a	2.342	0.002	0.115	0.226

^1^ Natural matter; ^2^ urea addition level (on a natural matter basis); ^3^ standard error of the mean; ^4^ means followed by different lowercase letters differ statistically (*p* < 0.05) according to Tukey’s test; ^5^ interaction effect between urea addition level and periods of aerobic exposure; ^6^ albumin:globulin ratio; ^7^ aspartate aminotransferase; ^8^ alanine aminotransferase; ^9^ gamma-glutamyl transferase.

## Data Availability

Not applicable.
